# Mapping the cancer research landscape across Zambia: evidence to support national cancer control planning

**DOI:** 10.3332/ecancer.2025.1942

**Published:** 2025-07-02

**Authors:** Susan Msadabwe, Peng Yun Ng, Richard Sullivan, Kennedy Lishimpi, John Kachimba, Justor Banda, Jane Mumba, Abidan Chansa, Mutuna Chiwele, Kasonde Bowa, Kaseya Chiyenu, Linda Malulu-Chiwele, Julie Torode, Grant Lewison, Andrew Leather, Ajay Aggarwal, Kathleen Schmeler, Groesbeck Parham, Kabisa Mwala, Paul Kamfwa

**Affiliations:** 1 Cancer Diseases Hospital, Lusaka, Zambia; 2 King’s College London, London, UK; 3 Cancer and Global Health, King’s College London, London, UK; 4 Conflict & Health Research Group; 5 Cancer Control, Ministry of Health, Lusaka, Zambia; 6 University Teaching Hospitals, Lusaka, Zambia; 7 Ndola Teaching Hospital, Ndola, Zambia; 8 Livingstone University Teaching Hospital, Livingstone, Zambia; 9 School of Medicine and Health Science, University of Lusaka, Lusaka, Zambia; 10 National Cancer Registry, Lusaka, Zambia; 11 Institute of Cancer Policy; 12 Global Health and Surgery, King’s College London, London, UK; 13 King’s Global Health Partnerships, London, UK; 14 Guy’s and St Thomas’ Trust; 15 Cancer System and Services, London School of Hygiene and Tropical Medicine, London, UK; 16 Gynecologic Oncology and Reproductive Medicine, The University of Texas MD Anderson Cancer Center, Houston, TX; 17 Women and Newborn Hospital, Zambia; a https://orcid.org/0000-0002-8186-0568; b https://orcid.org/0000-0002-6435-1825; c https://orcid.org/0000-0003-0500-5962; d https://orcid.org/0000-0001-9645-6659; e https://orcid.org/0000-0002-9670-4189; † Joint first authors

**Keywords:** cancer, global oncology, implementation science, research, health policy

## Abstract

**Background:**

Zambia faces the double burden of rising cancer incidence and a disproportionate volume of mortality from delayed presentations. The Ministry of Health Zambia acknowledged cancer research as a key pillar of cancer control in the National Cancer Control Strategic Plan 2022–2026, but there remains a paucity of country-specific evidence to inform strategies, implementation, monitoring and evaluation of research activities. Our study aimed to map and critically analyse the existing cancer research landscape to inform national planning.

**Methods:**

We adopted a two-stage mixed-method research. First, we conducted a systematic review, including 76 Zambian cancer studies published between 2012 and 2022, adhering to PRISMA guidance. Second, we conducted an in-person modified consensus meeting in Ndola, Zambia attended by 31 domestic and international stakeholders, to co-develop priorities and strategies based on gaps and facilitators identified through the systematic review.

**Results:**

The year-on-year cancer research output in Zambia had risen and diversified beyond cervical cancer but prevention, palliative care and health economic studies were lacking. Delay in deciding to seek care was most studied (n = 17, 63.0%), especially in cervical cancer. Research activities were mostly retrospective (*n* = 47/76, 61.8%) with only one randomised controlled trial identified. Greater than 90% (*n* = 10/11, 90.9%) of the most prolific research funders were international, predominantly from the United States and the United Kingdom, and Zambian researchers were under-represented as first and last authors at 43% (*n* = 33/76) and 45% (*n* = 34/76), respectively. The existing national cervical cancer registry, active global collaboration and adoption of technology were facilitators to be leveraged to build research capacity through multi-level, stakeholder-specific strategies.

**Conclusion:**

To strengthen research capacity, sustained commitment to priorities through the implementation of co-developed strategies is required at individual, organisational and institutional levels. This paradigm shift is necessary to deliver evidence-based cancer care tailored to the needs of Zambians with emphasis on value and quality.

## Introduction

Over the next 50 years, the global cancer burden is estimated to increase disproportionately in the low and middle-income countries (LMICs). The low-income countries are estimated to have the highest increase by 400%, followed by middle-income countries at 168%; and lastly, high-income countries (HICs) by 53% [1]. This translates into a projection of 75% of all cancer mortality LMICs by 2030 [2].

Zambia falls under low-income country and; thus, not precluded in this prognostication. The Global Cancer Observatory (GLOBOCAN) 2022 reported the country to be in the top ten of African countries with the greatest burden of cancer at an estimated age-standardised incidence rate and age-standardised mortality rate of 159.5 and 109.2 per 100,000 population, respectively [3]. Paired with low public awareness and the lack of access to cancer services nationwide, the country faces the twin burden of rising cancer incidence from a burgeoning population and a disproportionate volume of metastatic cancer from delayed presentations [6].

To overcome this challenge, cancer research is key to advance high quality cancer prevention, diagnosis and treatment options [7]. Yet, despite the high cancer burden faced by LMICs, less than 4% of the total global annual cancer research output as measured by publication came from authors from these countries [8]. In fact, African authors represent the smallest proportion of all LMICs [9]. Furthermore, the inequalities of cancer research do not only exist across continents but also within Africa, with only South Africa and Egypt contributing to 62% of all African cancer research [10].

To tackle the disparity in global cancer research, international stakeholders from the Global North could play important roles [7]. Research activities could be supported through the provision of funding as well as the sharing of research expertise and logistics with the aim of building research capacity [11]. In Zambia specifically, the country has received immense support from the governmental Cancer Moonshot initiative launched by the National Cancer Institute of the United States (US) [12]. It also maintains collaborations with international non-governmental institutions such as the International Agency for Research on Cancer (IARC), an arm of the World Health Organisation (WHO), International Atomic Energy Agency (IAEA), African Cancer Registry Network, African Organisation for Research and Training in Cancer (AORTIC) and Union for International Cancer Control (UICC), as well as partnerships with leading academic cancer centers such as MD Anderson Cancer Centre (MDA) and King’s College London (KCL) [17].

While international collaborations are often positive, their contribution is not always fair, sustainable or effective [11]. In fact, the funding chasm and the lack of local research leadership and capacity mean that the recipient country is often susceptible to power imbalances and research parachutism from the HICs. [20] A recent bibliometric review of global oncology publications found that among all studies conducted in and about sub-Saharan Africa, only 45% and 41% of the first and last authors, respectively, are African investigators [21]. More strikingly, of all clinical trials which enrolled patients from LMICS, only eight percent of all cancer-related randomised clinical trials are led by investigators from the LMICs, underlining the mismatch between global cancer burden and global cancer research leadership [22,23]. Such underrepresentation means that the local interests and priorities are more likely to be set by third parties, thus overlooked and understudied [24].

The National Cancer Control Strategic Plan (NCCSP) 2022–2026 released by the Ministry of Health Zambia (MOHZ) responded to this risk by prioritising the building of local cancer research capacity with the aid of international collaboration [25]. This goal is vital as the success of the Zambian cancer control plan will otherwise be limited by the paucity of country-specific evidence to inform implementation, monitoring and evaluation of cancer activities with an emphasis on quality, value and sustainability [7]. For example, the twin drivers of cancer specific to Zambia are the HIV and oncogenic HPV, resulting in cervical cancer and Kaposi sarcoma being the top two main contributors of cancer burden in the nation despite them being preventable [26–29]. These risk factors should be the focus of Zambian cancer research to generate local evidence to inform cancer control plans as opposed to other risk factors such as diet and smoking that could be more pertinent in other countries.

Henceforth, to overcome this challenge, we conducted this two-stage research involving a systematic review followed by a modified consensus meeting with domestic and international stakeholders to map and to analyse the cancer research landscape in Zambia. Our aim is to provide a high-resolution snapshot, backed by a thorough interrogation of the current practices, to identify gaps and facilitators in the current research ecosystem. They are utilised to set priorities and to design pragmatic, country-specific strategies to advance cancer research in Zambia.

## Materials and methods

We adopted a two-stage mixed-method research to map and investigate the current cancer research landscape in Zambia. The first stage is a systematic review and the second stage involves in-person modified consensus meetings involving domestic and international stakeholders.

### Stage 1: systematic review

A systematic review was carried out on 1st January 2023 using the PubMed and Web of Science database to include studies published between 1st January 2012 and 31st December 2022, following the PRISMA guidance. The search strategy is available in supplementary data (Appendix A).

### Inclusion criteria

Published full-text articles describing cancer specifically in Zambia were considered for inclusion. Articles must be in English language in a peer-reviewed journal published between the dates specified above.

### Exclusion criteria

Any articles not related to cancer in the context of Zambia and written in non-English language were excluded. Review articles, laboratory research, case reports, conference proceedings and repeated studies were also excluded.

### Data selection

Richard Sullivan (RS) carried out the initial search. Peng Yun Ng (PN) selected articles meeting the inclusion criteria from title and abstracts for full-text review. Subsequently, PN assessed the full-text articles to consider whether the inclusion and/or exclusion criteria were met.

### Data extraction

PN extracted data from all included studies, with consultation from RS and Ajay Aggarwal. Data extracted included:

- year of study publishedcharacteristics of study (research design, sample size, year of data collection)
- source of funding
- origin of first and last authorships
- data type (primary versus secondary)
- type of cancer
- cancer care pathway (prevention, aetiology, epidemiology, diagnostics, treatment or palliative care)
- whether study was exclusive to Zambia

For articles focussing on cancer diagnostics, they are further categorised using the three delays framework to identify indirect barriers to cancer diagnosis and treatment from onset of symptoms to rehabilitation after treatment, contributing to cancer morbidity and mortality [27]. The three delays are:

- delay in decision to seek care
- delay in identifying and reaching health facility for care
- delay in receiving quality care

### Stage 2: modified consensus meeting

A 1-day, in-person consensus meeting, themed ‘The Future of Cancer Research In Zambia’, was held on 8th January 2024 in Ndola, Zambia. It was organised by KCL and the MOHZ with support from MDA. The objectives of the meeting were to establish consensus in key areas of development and to co-develop priorities and strategies with local stakeholders to build cancer research capacity in Zambia. It was attended by 31 participants – 19 Zambian representatives, 8 from MDA and 4 from KCL.

The Zambian attendees were composed of eight surgeons of gynaecologic, breast, general and urological specialties, three physicians (two of whom specialise in palliative care), three nurses (two oncology nurse and one theatre nurse), one clinical oncologist, one pathologist, one cancer registry manager, one cancer support network community representative and one cancer patient representative. The international representatives are made up of experts in global oncology as well as cancer systems and services alongside surgical oncologists (gynaecology, breast and urology).

In terms of the format of the meeting, KCL started by informing all attendees of the key findings of the systematic review, focusing on the gaps and facilitators of cancer research in Zambia. Based on consensus, attendees from all institutions identified eight areas of development and discussed them in order of agreed priority to co-develop strategies. Following this, the attendees discussed the facilitators, primarily the research opportunities through international collaborations and existing databases, and highlighted nine areas of strength for further development. The meeting then concluded with the co-design of an NCCSP research committee and the prioritisation of their tasks upon the constitution.

## Results

### Systematic review

**Figure 1.**
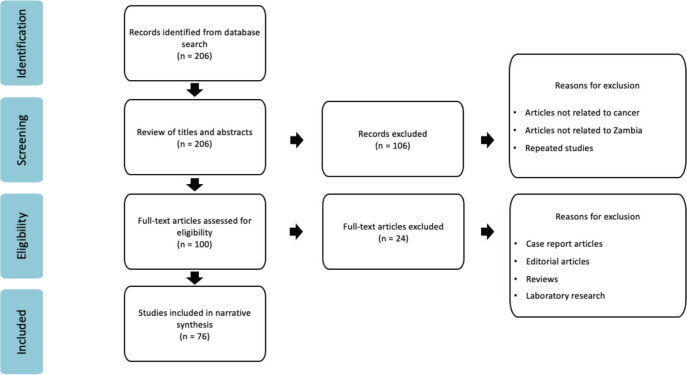
PRISMA flow chart of identification for articles for inclusion.

The initial search identified 206 articles. After all titles and abstracts were screened, 100 articles were chosen for full-text review. Of these, 76 studies were included for data extraction and analysis. The study selection process followed the PRISMA, as illustrated in Figure 1.

A summary of all the included publications, describing the spectrum of cancer research along the cancer care pathway in Zambia, is available as Appendix B.

### Cancer care pathway

The purpose of research is synthesised in six stages – prevention, aetiology, epidemiology, diagnostics, treatment and palliative care – to make up the cancer care pathway.

**Figure 2.**
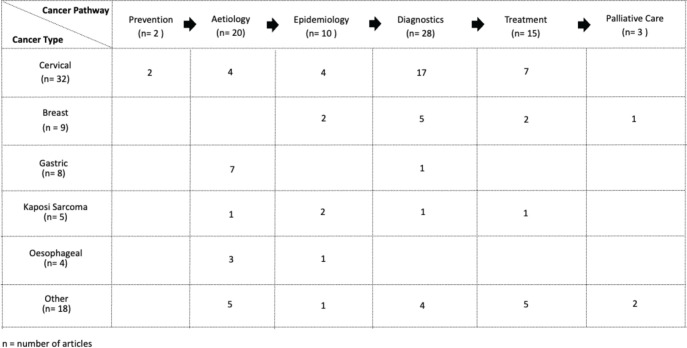
Swimlane diagram of the cancer care pathway by cancer site in Zambia.

Cancer research in Zambia predominantly focussed on diagnostics (*n* = 28, 36.8%), [5,6,53] followed by aetiology (*n* = 21, 27.6%) [73] and delivery of cancer treatment (*n* = 15, 19.7%) [85]. Epidemiology (*n* = 10, 13.2%) [94], palliative care (*n* = 3, 3.9%) [96] and prevention (*n* = 2, 2.6%) [45] lagged behind in descending order. The cancer care pathway, categorised by purpose and cancer type, is illustrated as a swim-lane diagram in Figure 2.

### Cancer type

**Figure 3.**
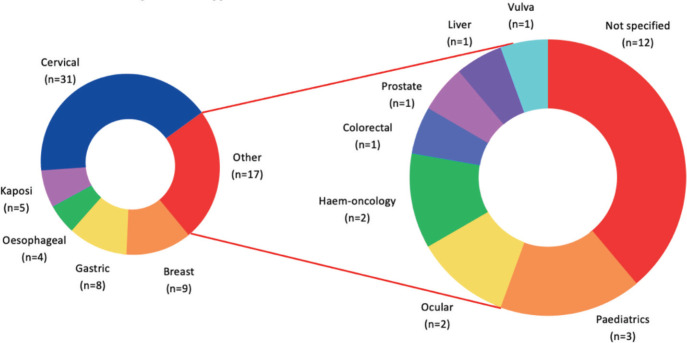
Cancer research by cancer type in Zambia.

Cervical cancer (*n* = 32, 42.1%) [6,26,28–36,38,42,44–46,50,52,63,57,58,66,75,78,80–84,86,88,93] emerged as the most studied cancer type, especially in its diagnosis (*n* = 17, 22.14%) [6,28–36,38,42,44–46,50,52]. Breast cancer (*n* = 9, 11.8%) [5,37,40,49,51,90,94,95,97], gastric cancer (*n* = 8, 10.5%) [43,54,55,59,64,67,68,70] and Kaposi sarcoma (*n* = 5, 6.6%) [48,61,76,87,89] in turn ranked second, third and fourth, respectively, in popularity. It is worth noting that there were four studies on oesophageal cancer (*n* = 4, 5.3%) [56,60,91,73], while there were only three studies on paediatrics (*n* = 3, 3.9%) [39,74,77], two studies on haem-oncology (*n* = 2, 2.6%) [62,65] and ocular cancers (*n* = 2, 2.6%) [69,71] and one study each for prostate (*n* = 1, 1.3%) [47], colorectal (*n* = 1, 1.3%) [41], liver (*n* = 1, 1.3%) [53] and vulva cancer (*n* = 1, 1.3%) [72]. The breakdown of cancer types researched in Zambia is detailed in the form of a pie chart in Figure 3.

### Trend of research

**Figure 4.**
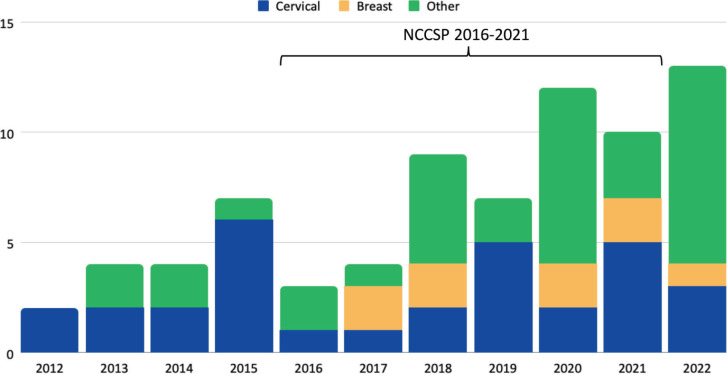
Cumulative cancer research output by cancer type from Zambia between 2012 and 2022.

**Figure 5.**
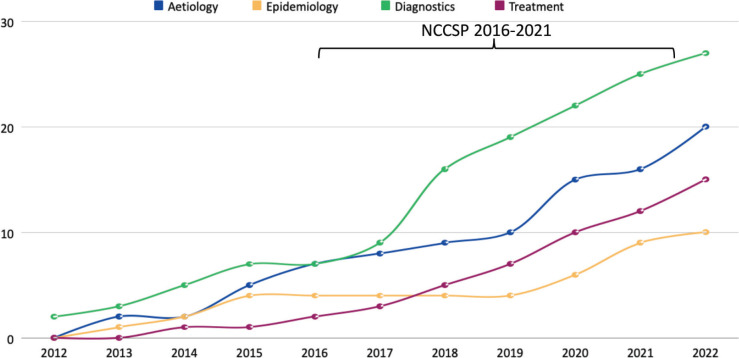
Cumulative cancer research output from Zambia by cancer care pathway.

The timeframe for the inclusion of studies was set to ensure the applicability and relevance of findings to the Zambian cancer care services that are rapidly evolving with the introduction of NCCSP 2016-2021. As such, we plotted the volume of cancer research output by cancer type and purpose across the decade as a histogram (Figure 4) and line graph (Figure 5), respectively, to zero in on the direction of travel with the aim to identify emerging trend or lack thereof within the healthcare system.

With cervical cancer being the initial focus of cancer research, we observed an increase in the variety of cancer types being studied across the years, prominently so for breast cancer, which surfaced as a topic of interest since 2017 (Figure 4).

In terms of the purpose of cancer research, diagnostics consistently gathered the most academic interest through the decade. It is closely followed by aetiology and treatment, which became increasingly popular from 2017 onwards and surpassed epidemiology in 2018 as the third most common purpose of cancer research (Figure 5). On the contrary, it is worth noting that there was a dearth of evidence in prevention and palliative care cancer research and an absence of health economics studies.

### Authorship and funding of research

Only 43% (*n* = 33/76) and 45% (*n* = 34/76) of the first and last authors of all studies in and around Zambia were based in Zambia.

On the other hand, most of the cancer research in Zambia is supported by international organisations. The US government, through the National Institute of Health and the National Cancer Institute, stood out as the most and second most prolific international funder to Zambian cancer research, respectively. Susan G Komen Foundation, a non-profit, non-governmental organisation focusing on breast cancer diagnosis and treatment ranked third; and the Zambian Ministry of Health and Ministry of Education, being the only local institution, ranked fourth. Seven out of 11 of the most prolific funders for cancer research in Zambia were American organisations; two - namely the UK Research and Innovation and the Wellcome Trust were British; and one, University Chinese Academy of Science, was Chinese.

The top ten most prolific cancer research funders in Zambia are summarised in Table 1.

### Three delays model

Among the articles which focused on cancer diagnostics, we utilised the three-delays model to seek insight into the cancer diagnosis and treatment. The three delays share interconnection. Henceforth, we identified them with the hope to shed light on the deficiency currently and guide cancer research moving forward.

**Figure 6.**
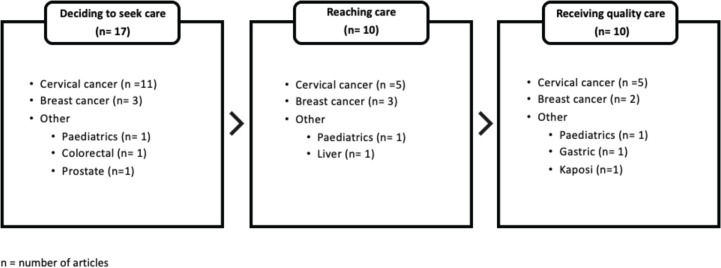
The three delays model for cancer diagnosis in Zambia.

Delay 1, deciding to seek care, was most prominent (*n* = 17, 63.0%) [5,29,30,32,36,38–42,44–47,49,50,52]; followed by Delay 2, deciding to reach care (*n* = 10, 37.0%) [28,33,34,37–39,49–51,53] and Delay 3, receiving quality care (*n* = 10, 37.0%) [6,31,34,35,37–39,43,48,49] consecutively. This is further broken down based on cancer type in Figure 6.

### Modality and research design of cancer treatment

In terms of research on cancer treatment, surgery (*n* = 6, 40%) [78, 80,82,84,85] is the mainstay modality. Chemotherapy (*n* = 2, 13.3%) [75,76] and radiotherapy (*n* = 2, 13.3%) [75,79] fell behind and were only first published in 2016. Other general modalities of treatment (*n* = 6, 40%) [37,39,49,74,77,81] explored include the use of interactive systems to facilitate multidisciplinary cancer care, complications and morbidities of cancer treatment, as well as efficiency of cancer treatment service provision.

**Figure 7.**
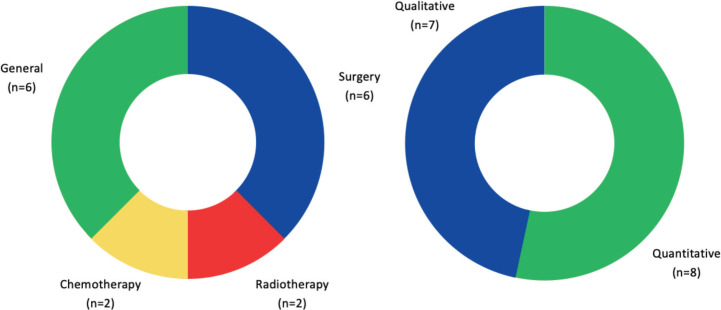
Cancer research by treatment modality and research design in Zambia.

The majority of the research design of cancer treatment is quantitative (*n* = 8, 53.3%) [37,49,74–76,78,81,83] and the remaining is qualitative (*n* = 7, 46.7%) [39,77,79,80,82,84,85] as shown in Figure 7. Ten of them were prospective observational studies [42,52,78,80–85,87]; four, retrospective observational studies [76,77,79,89]; and one, randomised controlled trial [83].

### Modified consensus meeting

#### Areas of development

Upon identifying the gaps in the cancer research activity in Zambia and the lack of independent cancer research led by local academics, the attendees came to a consensus of eight key areas of development to build sustainable cancer research capacity in Zambia. The key areas of development in no specific order were as follows:

1. Improving research writing skills
2. Building name recognition of local academics
3. Increasing domestic funding
4. Improving routine data collection for research, audit and quality improvement
5. Improving access to publication through local journals
6. Encouraging mentorship in research
7. Setting research priorities
8. Providing workforce incentives for research

#### Areas of strength

With the aim of building a strong and sustainable cancer research ecosystem in Zambia, the attendees identified international collaboration as leverage and nine areas of strength of the existing international research opportunities to focus on:

1. Ensuring research is solution-based and tailored to the need of Zambia
2. Aligning research with and contributing to the NCCSP target
3. Underpinning research with rigorous literature review
4. Building supporting infrastructure through technology, i.e., virtual tumour board and project ECHO
5. Promoting individual research capacity building through mentorship
6. Strengthening research design skills
7. Partnering with established local research institutions such as ZAMBART
8. Diversifying research methodologies
9. Collaborating with international partners in the development of research proposals

#### The NCCSP research committee

The constitution of a cancer control research committee was identified through consensus as key to guide and shape future review and decision making for research in cancer prevention and control in Zambia. The committee should represent the nation’s oncology community and provide expert advice on the subject matter to the governmental authorities. The four key questions for the committee to consider were presented as followed:

1. How do we shift the cancer research narrative to one of home-grown research priorities?
2. How do we ensure we are marrying research with the current cancer burden and educational needs of the country?
3. How do we build in research infrastructure, such as study administrative support and laboratory needs?
4. How do we build new sustainable research programmes accessible to local clinicians?

## Discussion

At the time of this review, Zambia was undergoing a process of decentralising its cancer services from the only Cancer Diseases Hospital in Lusaka, as set out in the NCCSP 2022–2026 [25]. Emphasis was put on cancer research with the hope that Zambian-specific data could be leveraged to inform cancer control planning, thus achieving better, more affordable and equitable outcomes for Zambian cancer patients. Our mixed-method research served to understand the existing research landscape so that within the limits of resource capability and capacity, cancer research could be prioritised based on nation’s health needs and optimised with strategies that leverage on the existing opportunities such as the national cervical cancer registry and strong international collaboration.

### Trends

Between 2012 and 2022, there is an overall rise in cancer research output in and of Zambia. Cancer research had also diversified beyond cervical cancer into breast cancer, gastric cancer, Kaposi Sarcoma and others since 2017 (Figure 4). Both of these trends were healthy indicators of the easiness of research in Zambia. One could infer from this phenomenon that the national prioritisation of multiple cancer types through the previous NCCSP 2016–2021 had an indirect effect on the type of research being conducted in the ecosystem. The national targets had also likely helped align funders’ interest so monetary resource was channeled in a coordinated fashion to solution-based research aligned with national priorities.

Given that Zambia faced the twin health burden of rising cancer incidence secondary to a burgeoning population and advanced (metastatic) cancer from delayed presentation, our review utilised the three-delays model to analyse the root causes. Upon dissection of the cancer diagnostic and treatment research, we found that this research primarily focused on the delay to decide to seek care (*n* = 17/27, 63.0%), especially for cervical cancer (*n* = 11/17, 65%), alluding to a knowledge gap in understanding the multi-factorial barriers that hinder the community from deciding to seek care. This finding was relevant as it provided a better understanding of the health behaviour and sociocultural norms which could be utilised to promote the early presentation and participation in cancer screening in Zambia. It formed the contextual evidence that are valuable in informing future national cancer control plans. More importantly, it is an exemplar for how research done based on priorities set through NCCSP could produce research output which in return informs further planning, thus propelling a virtuous cycle.

### Gaps

A recent review on the African cancer research ecosystem, by Rubagumya *et al* [97], demonstrated the vicious cycle propelling disparity in cancer research through stages including where, how, which, by whom and by whose funding cancer research was done in. Our review highlighted similar vulnerability in the Zambian research ecosystem. Notably, there is a lack of diversity in where and how research was conducted. The majority of research was either based in Lusaka, namely in the Cancer Diseases Hospital and the University Teaching Hospital or was reliant on data from the single national cervical cancer registry. This gap highlights the need to implement a strategy for cancer research as part of the decentralisation effort to buttress the research culture nationwide and to produce high-quality evidence that are truly representative of Zambia, instead of being low-quality and urban-biased. Furthermore, there is a general lack of breadth and quality in cancer research produced in Zambia. Most publications were limited to observational studies which were retrospective (*n* = 47/76, 61.8%). There were also a paucity of randomised clinical trials (*n* = 1/76, 1.3%), to validate cancer treatment options for Zambians, and an absence of health macro- and micro-economics studies, which are vital to capture economic, welfare and social values of cancer care [98]. This phenomenon aligned with the rest of the LMICs as a recent review revealed that only a meagre 8%–14% of published economic evaluations of health interventions are from those countries [99]. However, these analyses are especially relevant in Zambia with the recent rollout of the National Health Insurance Scheme in 2018 which embraces cancer care [100]. They are key to inform health financing decision-making on a populational level.

In terms of the key investigators of cancer research in and about Zambia, only 43% (*n* = 33/76) and 45% (*n* = 34/76) of first and last authors, respectively, are local. This is in accordance with a bibliometric review of studies in sub-Saharan Africa that found that African investigators only made up 45% and 41% of first and last authorship of publications, respectively [21]. Few investigators stood out as high-output individuals, reflecting the concentration of academic pursuit primarily in individuals with name recognition. Most studies with international authors were the results of a handful of old collaborations established through grants. More importantly, more than 90% (*n* = 10/11) of the most prolific research funders were international, predominantly from the US (Table 1). These findings exposed the vulnerability of the researchers in Zambia to power imbalances and research parachutisms, which could undermine their autonomy and motivation in research, thus eroding local research capacity in the long run.

### Facilitators

On the contrary, the existing national cervical cancer registry, active global collaboration and adoption of technology were highlighted in our systematic review and the modified consensus meeting as the facilitators that Zambian could leverage to expand cancer research capacity sustainably. While Zambia only has one operational population-based cancer registry (PBCR) in Lusaka, this registry has served as a valuable databank that underpinned screening practices nationwide and contributed to multiple high-quality publications specific to the country. Such effort should be replicated in the process of decentralising cancer services.

Second, global collaboration was a major asset to the country as beyond funding, it nurtured local clinicians through knowledge and skills transfer. For example, 36% (*n* = 27/76) of the publications had a mixed of Zambian and non-Zambian first and last authors. This cross-pollination of research practices helped build relevant research skills in local academics and keep them motivated as they pursue research tailored to the need of their clinical environment. The Zambian government should maintain and strengthen international collaborations through bilateral agreements. A positive example of this was the recent signing of a memorandum of understanding between the MOHZ and MDA cancer centre, which served to bolster their partnership over the next 5 years [17]. Efforts to expand collaborations should also be encouraged as there are other key non-governmental stakeholders such as the UICC, IAEA and WHO which are in charge of international research collaborations. The success of the African Breast Cancer- Disparity in Outcome (ABC-DO) cohort study which included Zambia as one of the five included countries in the continent was laudable and was testimony of such partnerships [90]. AORTIC, which conducted the Choose Wisely Africa consensus meeting to develop guidelines tailored to the healthcare capacities, infrastructure and sociocultural factors in African countries including Zambia is another exemplar, driving solution-based research that emphasises on value and quality of cancer care [101].

Third, the adoption of technology in cancer research across different clinical settings, such as the virtual tumour board for telepathology and teleradiology, point-of-care diagnostics and low-cost virtual reality to build surgical oncology capacity, were promising facilitators of cancer research. Most technology helped drive research not only in the process of integrating into clinical practice when new practices were validated, but also after they were fully integrated when the process of data collection, storage and analysis was standardised and automated, forming a valuable databank. It is very likely that the procurement and introduction of nationwide information and imaging systems, such as the Hospital Incident Command System and the Picture Archiving and Communication System, as planned in the NCCSP 2022–2026, will transform, beyond clinical care, the cancer research landscape in Zambia over the next decades.

### Priorities and strategies

The effort to strengthen cancer research capacity in Zambia should be at both individual and system levels to ensure long-term sustainability. The UK Department for International Development successfully adopted the three-level model, tiered by individual, organisational and institutional stakeholders to develop and decentralise research capacity [102]. The model helped continuously engage with various stakeholders in capacity assessment, strategising and planning, implementation, monitoring and evaluation.

Henceforth, in this review, we adopted the same approach to identify priorities and develop cancer research capacity-building strategies applicable to Zambia (Table 2).

### Strength and limitation

The strengths of our research lie in the two-stage mixed-method approach we adopted. It allowed us to not only map but also co-analyse the cancer research landscape with local stakeholders using the consensus approach. This unique approach facilitated early buy-in from various local stakeholders, encouraging them to take ownership of the identified problems, set priorities and implement the co-developed strategies.

The limitations of our research are in the non-inclusion of non-English studies, studies published before 2012, unpublished literature or reports from cancer advocacy groups, agencies or registries and studies published in the African Index Medicus thus not included in the PubMed or Web of Science repositories.

## Conclusion

To relieve the spiralling cancer burden in Zambia, cancer research is key to the cancer control plan. While cancer research activity had risen in the past decade, prevention, palliative care and health economic studies were generally lacking. Our critical analysis further exposed the risk of power imbalances and research parachutism in the current research ecosystem due to an overwhelming dependence on monetary and non-monetary support from international collaborations.

To truly decolonise the research ecosystem, research capacity needs to be strengthened at individual, organisational and institutional level, leveraging on existing facilitators including experience with PBCR databases, international collaborations and adoption of technology. We co-identified priorities and co-developed strategies with domestic and international stakeholders to inform future cancer control plans. Ultimately, this paradigm shift will require sustained commitment through the stages of strategising, implementation, monitoring and evaluation of the plan to deliver value and quality cancer care tailored to the need of Zambians.

## Conflicts of interest

The authors have no conflicts of interest to declare.

## Figures and Tables

**Table 1.** Top ten most prolific cancer research funders in Zambia.

Rank	Name of Funder	Number of Studies Funded
1	Fogarty International Centre, National Institute of Health U.S.	26
2	National Cancer Institute U.S.	19
3	Susan G Komen Foundation	7
4	Ministry of Health, Ministry of Education, Zambia	5
5	U.S. President's Emergency Plan for AIDS Relief (PEPFAR)	4
	Wellcome Trust	4
7	American Relief and Recovery Act	3
	National Institute of Allergy and Infectious Disease U.S.	3
	U.S Civilian Research & Development Foundation (CRDF Global)	3
10	University of Chinese Academy of Sciences (UCAS)	2
	MD Anderson Cancer Center	2
	U.K. Research and Innovation	2

**Table 2.** Cancer research capacity-building priorities and strategies tiered by individual, organisational and institutional stakeholders.

	Stakeholders	Priorities	Strategies
Individual	International researchers	To mentor Zambian researchers with the intent to transfer research skillsets and to build accreditation while respecting their autonomy To improve Zambian researchers writing skills by providing feedback and guidance To understand the difficulties faced by Zambian clinicians to engage in research, i.e., lack of study administrative support, protected time, and formal training opportunities	To implement structured reflexivity statements and transparency matrix To offer fellowship and post-graduate research degrees for Zambian-based research To build a cancer research team to offer administrative support to Zambian researchers To integrate research into medical training curriculum and offer protected time for academic clinicians
	Zambian researchers		
Organisational	Local journals (i.e., Medical Journal of Zambia) International journals (i.e., New England Journal of Medicine)	To deal with the prohibitive cost of publication which discentivises Zambian researchers To build profile and recognition of local journals To encourage clinicians’ involvement and submissions throughout medical training	To waive publication fees for Zambian research (similar to the WHO Hinari system for Middle Eastern LMICs) [103] To establish a journal club with training opportunities to gain following while train local reviewers To form partnerships with more established journals to create publication opportunities in high-impact journals
	Local non-governmental organisation (i.e., ZAMBART, Zambian Cancer Society, Teal Sisters) International non-governmental organisations (i.e., WHO, AORTIC, IAEA, UICC) International academic institutions (i.e., MDA, KCL, UCAS, Vanderblitz)	To promote patient-public involvement in cancer research to improve patient experience and promote awareness To facilitate south-south collaborations for research initiatives concerning cancer care To build local research capacity in research design, protocol development and grant writing To improve access to funding for Zambian-based research taking into consideration the challenges local investigators faced	To engage with trusted civil societal partners in cancer health promotion and research focusing on cancer patient experience using patient reported outcome measures (PROMs) To design and conduct international research collaboratives, such as the ABC-DO cohort study, involving investigators from across the African continent To run skill-based research workshop focussing on various research skillsets for Zambian researchers To offer research grants that are designed with specific eligibility criteria suitable for African researchers To organise international and national scientific meetings to promote dissemination of research findings and collaboration
	International non-governmental organisatons (Susan G Komen, Wellcome Trusts) Foreign governmental institutions (i.e., NCI, PEPFAR, UKRI)		
Institutional	Ministry of Health Zambia	To set up regulatory and legislative frameworks and bodies to strategize, implement, monitor and evaluate collaborative cancer research To leverage the success of national cervical cancer registry to expand the utilisation of PBCR To improve access to domestic funding for cancer research	To establish cancer research centre of excellence To prioritise the constitution of a NCCSP research committee and a local ethics committee To fund population-based cancer-specific registries To commit to boosting internal funding for cancer research through NCCSP and appropriate health finance budgeting
	National Health Research Authority (NHRA)	To include cancer research as an integral part of the NHRA and offer support and guidance accordingly	To allocate monetary and non-monetary resources for cancer research in the budget

## Supplementary materials

### 1. Appendix A. Search strategies.

Database: PubMed and Web of Science database (01/01/2012 and 31/12/2022)

Date of search: 01/01/2023

Step search term details

1 [ONCOL filter] Applied to retrieve oncology literature

2 CU=Zambia Country of publication limited to Zambia

ONCOL filter

SO=(ACTA-ONCOLOGICA OR ADVANCES-IN-CANCER-BIOMARKERS-FROM-BIOCHEMISTRY-TO-CLINIC-FOR-A-CRITICAL-REVISION OR ADVANCES-IN-CANCER-RESEARCH OR ADVANCES-IN-IMMUNOLOGY OR AMERICAN-JOURNAL-OF-CANCER-RESEARCH OR AMERICAN-JOURNAL-OF-CLINICAL-ONCOLOGY-CANCER-CLINICAL-TRIALS OR ANNALS-OF-ONCOLOGY OR ANNALS-OF-SURGICAL-ONCOLOGY OR ANTI-CANCER-AGENTS-IN-MEDICINAL-CHEMISTRY OR ANTI-CANCER-DRUGS OR ANTICANCER-RESEARCH OR APPLICATIONS-OF-VIRUSES-FOR-CANCER-THERAPY OR ASIAN-PACIFIC-JOURNAL-OF-CANCER-PREVENTION OR ASIA-PACIFIC-JOURNAL-OF-CLINICAL-ONCOLOGY OR BIOCHIMICA-ET-BIOPHYSICA-ACTA-REVIEWS-ON-CANCER OR BIOLOGICAL-BASIS-OF-ALCOHOL-INDUCED-CANCER OR BLOOD-CANCER-JOURNAL OR BMC-CANCER OR BRAIN-TUMOUR-PATHOLOGY OR BREAST-CANCER OR BREAST-CANCER-RESEARCH OR BREAST-CANCER-RESEARCH-AND-TREATMENT OR BRITISH-JOURNAL-OF-CANCER OR BULLETIN-DU-CANCER OR CA-A-CANCER-JOURNAL-FOR-CLINICIANS OR CANCER OR CANCER-AND-METASTASIS-REVIEWS OR CANCER-BIOLOGY-THERAPY OR CANCER-BIOMARKERS OR CANCER-BIOTHERAPY-AND-RADIOPHARMACEUTICALS OR CANCER-CAUSES-CONTROL OR CANCER-CELL OR CANCER-CELL-INTERNATIONAL OR CANCER-CHEMOTHERAPY-AND-PHARMACOLOGY OR CANCER-CONTROL OR CANCER-CYTOPATHOLOGY OR CANCER-DISCOVERY OR CANCER-EPIDEMIOLOGY OR CANCER-EPIDEMIOLOGY-BIOMARKERS-PREVENTION OR CANCER-GENE-THERAPY OR CANCER-GENETICS OR CANCER-GENOMICS- PROTEOMICS OR CANCER-IMAGING OR CANCER-IMMUNOLOGY-IMMUNOTHERAPY OR CANCER-IMMUNOLOGY-RESEARCH OR CANCER-INVESTIGATION OR CANCER-JOURNAL OR CANCER-LETTERS OR CANCER-MEDICINE OR CANCER-NURSING OR CANCER-PREVENTION-RESEARCH OR CANCER-RADIOTHERAPIE OR CANCER-RESEARCH OR CANCER-RESEARCH-AND-TREATMENT OR CANCER-SCIENCE OR CANCER-TREATMENT-REVIEWS OR CARCINOGENESIS OR CELL-POLARITY-AND-CANCER OR CELLULAR-ONCOLOGY OR CHEMOTHERAPY OR CHINESE-JOURNAL-OF-CANCER OR CHINESE-JOURNAL-OF-CANCER-RESEARCH OR CLINICAL-&-EXPERIMENTAL-METASTASIS OR CLINICAL-BREAST-CANCER OR CLINICAL-CANCER-RESEARCH OR CLINICAL-COLORECTAL-CANCER OR CLINICAL-GENITOURINARY-CANCER OR CLINICAL-JOURNAL-OF-ONCOLOGY-NURSING OR CLINICAL-LUNG-CANCER OR CLINICAL-LYMPHOMA-MYELOMA-LEUKEMIA OR CLINICAL-ONCOLOGY OR CLINICAL-TRANSLATIONAL-ONCOLOGY OR CRITICAL-REVIEWS-IN-ONCOLOGY-HEMATOLOGY OR CURRENT-ADVANCES-IN-OSTEOSARCOMA OR CURRENT-CANCER-DRUG-TARGETS OR CURRENT-ONCOLOGY OR CURRENT-ONCOLOGY-REPORTS OR CURRENT-OPINION-IN-ONCOLOGY OR CURRENT-PROBLEMS- IN-CANCER OR CURRENT-TREATMENT-OPTIONS-IN-ONCOLOGY OR ENDOCRINE-RELATED-CANCER OR EUROPEAN-JOURNAL-OF-CANCER OR EUROPEAN-JOURNAL-OF-CANCER-CARE OR EUROPEAN-JOURNAL-OF-CANCER-PREVENTION OR EUROPEAN-JOURNAL-OF-GYNAECOLOGICAL-ONCOLOGY OR EUROPEAN-JOURNAL-OF-ONCOLOGY OR EUROPEAN-JOURNAL-OF-ONCOLOGY-NURSING OR EXPERT-REVIEW-OF-ANTICANCER-THERAPY OR FAMILIAL-CANCER OR FUTURE-ONCOLOGY OR GASTRIC- CANCER OR GENES-CHROMOSOMES-CANCER OR GUIDANCE-MOLECULES-IN-CANCER-AND-TUMOUR-ANGIOGENESIS OR GYNECOLOGIC-ONCOLOGY OR HEAD-NECK-ONCOLOGY OR HEMATOLOGICAL-ONCOLOGY OR HEMATOLOGY-ONCOLOGY-CLINICS-OF-NORTH-AMERICA OR HEREDITARY-CANCER-IN-CLINICAL-PRACTICE OR HORMONES-CANCER OR IMMUNITY-TO-LISTERIA-MONOCYTOGENES OR INDIAN-JOURNAL-OF-CANCER OR INFECTIOUS-AGENTS-AND-CANCER OR INFLAMMATION-AND-CANCER OR INTEGRATIVE-CANCER-THERAPIES OR INTERNATIONAL-JOURNAL-OF-CANCER OR INTERNATIONAL-JOURNAL-OF-CLINICAL-

ONCOLOGY OR INTERNATIONAL-JOURNAL-OF-GYNECOLOGICAL-CANCER OR INTERNATIONAL-JOURNAL-OF-ONCOLOGY OR INTERNATIONAL-JOURNAL-OF-RADIATION-ONCOLOGY-BIOLOGY-PHYSICS OR JAPANESE-JOURNAL-OF-CLINICAL-ONCOLOGY OR JNCI-JOURNAL-OF-THE-NATIONAL-CANCER-INSTITUTE OR JOURNAL-OF-ADOLESCENT-AND-YOUNG-ADULT-ONCOLOGY OR JOURNAL-OF-BONE-ONCOLOGY OR JOURNAL-OF-BREAST-CANCER OR JOURNAL-OF-CANCER OR JOURNAL-OF-CANCER-EDUCATION OR JOURNAL-OF-CANCER-RESEARCH-AND-CLINICAL-ONCOLOGY OR JOURNAL-OF-CANCER-RESEARCH-AND-THERAPEUTICS OR JOURNAL-OF-CANCER-SURVIVORSHIP* OR JOURNAL-OF-CHEMOTHERAPY OR JOURNAL-OF-CLINICAL-ONCOLOGY OR JOURNAL-OF-ENVIRONMENTAL-PATHOLOGY-TOXICOLOGY-AND-ONCOLOGY OR JOURNAL-OF-EXPERIMENTAL-CLINICAL-CANCER-RESEARCH OR JOURNAL-OF-GERIATRIC-ONCOLOGY OR JOURNAL-OF-GYNECOLOGIC-ONCOLOGY OR JOURNAL-OF-HEMATOLOGY-ONCOLOGY OR JOURNAL-OF-MEDICAL-IMAGING-AND-RADIATION-ONCOLOGY OR JOURNAL-OF-NEURO-ONCOLOGY OR JOURNAL-OF-PEDIATRIC-HEMATOLOGY-ONCOLOGY OR JOURNAL-OF-PEDIATRIC-ONCOLOGY-NURSING OR JOURNAL-OF-PSYCHOSOCIAL-ONCOLOGY OR JOURNAL-OF-SURGICAL-ONCOLOGY OR JOURNAL-OF-THE-NATIONAL-CANCER-INSTITUTE OR JOURNAL-OF-THE-NATIONAL-COMPREHENSIVE-CANCER-NETWORK OR JOURNAL-OF-THORACIC-ONCOLOGY OR LANCET-ONCOLOGY OR LEUKEMIA OR LEUKEMIA-LYMPHOMA OR LEUKEMIA-RESEARCH OR LUNG-CANCER OR MEDICAL-ONCOLOGY OR MELANOMA-RESEARCH OR MICRORNA-CANCER-FROM-MOLECULAR-BIOLOGY-TO-CLINICAL-PRACTICE OR MOLECULAR-CANCER OR MOLECULAR-CANCER-RESEARCH OR MOLECULAR-CANCER-THERAPEUTICS OR MOLECULAR-CARCINOGENESIS OR MOLECULAR-ONCOLOGY OR NATURE-REVIEWS-CANCER OR NATURE-REVIEWS-CLINICAL-ONCOLOGY OR NEOPLASIA OR NEOPLASMA OR NEUROENDOCRINE-TUMOURS-A-MULTIDISCIPLINARY-APPROACH OR NEURO-ONCOLOGY OR NUTRITION-AND-CANCER-AN-INTERNATIONAL-JOURNAL OR ONCOGENE OR ONCOGENESIS OR ONCOIMMUNOLOGY OR ONCOLOGIE OR ONCOLOGIST OR ONCOLOGY OR ONCOLOGY-LETTERS OR ONCOLOGY-NEW-YORK OR ONCOLOGY-NURSING-FORUM OR ONCOLOGY-REPORTS OR ONCOLOGY-RESEARCH OR ONCOLOGY-RESEARCH-AND-TREATMENT OR ONCOTARGET OR ONCOTARGETS-AND-THERAPY OR ONKOLOGE OR ONKOLOGIE OR ORAL-ONCOLOGY OR PATHOLOGY-ONCOLOGY-RESEARCH OR PEDIATRIC-BLOOD-CANCER OR PEDIATRIC-HEMATOLOGY-AND-ONCOLOGY OR PIGMENT-CELL-MELANOMA-RESEARCH OR PROGRESS-IN-TUMOUR-RESEARCH OR PROSTATE-CANCER-AND-PROSTATIC-DISEASES OR PSYCHO-ONCOLOGIE OR PSYCHO-ONCOLOGY OR RADIATION-ONCOLOGY OR RADIOLOGY-AND-ONCOLOGY OR RADIOTHERAPY-AND-ONCOLOGY OR RECENT-PATENTS-ON-ANTI-CANCER-DRUG-DISCOVERY OR RENAISSANCE-OF-CANCER-IMMUNOTHERAPY OR SEMINARS-IN-CANCER-BIOLOGY OR SEMINARS-IN-ONCOLOGY OR SEMINARS-IN-RADIATION-ONCOLOGY OR STRAHLENTHERAPIE-UND-ONKOLOGIE OR SUCCESSES-AND-LIMITATIONS-OF-TARGETED-CANCER-THERAPY OR SUPPORTIVE-CARE-IN-CANCER OR SURGICAL-ONCOLOGY-CLINICS-OF-NORTH-AMERICA OR SURGICAL-ONCOLOGY-OXFORD OR TARGETED-ONCOLOGY OR TECHNOLOGY-IN-CANCER-RESEARCH-TREATMENT OR THERAPEUTIC-ADVANCES-IN-MEDICAL-ONCOLOGY OR THORACIC-CANCER OR TRANSLATIONAL-ONCOLOGY OR TUMOUR-BIOLOGY OR TUMORI OR TUMOUR-MICROENVIRONMENT-AND-CELLULAR-STRESS-SIGNALING-METABOLISM-IMAGING-AND-THERAPEUTIC-TARGETS OR UHOD-ULUSLARARASI-HEMATOLOJI-ONKOLOJI-DERGISI OR UROLOGIC-ONCOLOGY-SEMINARS-AND-ORIGINAL-INVESTIGATIONS OR VETERINARY-AND-COMPARATIVE-ONCOLOGY OR WORLD-JOURNAL-OF-SURGICAL-ONCOLOGY OR WSPOLCZESNA-ONKOLOGIA-CONTEMPORARY-ONCOLOGY OR ADVANCES-IN-RADIATION-ONCOLOGY OR ADVANCES-IN-RESPIRATORY-CANCEROGENESIS OR AGING-CANCER-AND-AGE-RELATED-DISEASES-COMMON-MECHANISM OR ALCOHOL-AND-CANCER OR AMERICAN-JOURNAL-OF-HEMATOLOGY-ONCOLOGY OR ANNUAL-REVIEW-OF-CANCER-BIOLOGY* OR ANTICANCER-GENES OR APOPTOSIS-IN-CANCER-PATHOGENESIS-AND-ANTI-CANCER-THERAPY-NEW-PERSPECTIVES-AND-OPPORTUNITIES OR APPROACHES-TO-UNDERSTANDING-BREAST-CANCER OR ASIA-PACIFIC-JOURNAL-OF-ONCOLOGY-NURSING OR ASIAN-ONCOLOGY-NURSING OR BIOLOGICAL-MECHANISMS-OF-MINIMAL-RESIDUAL-DISEASE-AND-SYSTEMIC-CANCER OR BIOMECHANICS-IN-ONCOLOGY OR BLADDER-CANCER OR BLOOD-AND-LYMPHATIC-CANCER-TARGETS-AND-THERAPY OR BREAST-CANCER-BASIC-AND-CLINICAL-RESEARCH OR BREAST-CANCER-MANAGEMENT OR BREAST-CANCER-METASTASIS-AND-DRUG-RESISTANCE-CHALLENGES-AND-PROGRESS-2ND-EDITION OR BREAST-CANCER-TARGETS-AND-THERAPY OR CANCER-AND-DEVELOPMENT OR CANCER-AND-ZEBRAFISH-MECHANISMS-TECHNIQUES-AND-MODELS OR CANCER-BIOLOGY-AND-THE-NUCLEAR-ENVELOPE-RECENT-ADVANCES-MAY-ELUCIDATE-PAST-PARADOXES OR CANCER-BIOLOGY-MEDICINE OR CANCER- COMMUNICATIONS OR CANCER-FORUM OR CANCER-GENETICS-AND-CYTOGENETICS OR CANCER-GROWTH-AND-METASTASIS OR CANCER-IMMUNOLOGY-AND-IMMUNOTHERAPY OR CANCER-IMMUNOTHERAPY OR CANCER-INFORMATICS OR CANCER-MANAGEMENT-AND-RESEARCH OR CANCER-METABOLISM OR CANCER-MICROENVIRONMENT OR CANCER-NANOTECHNOLOGY

OR CANCER-REPORTS OR CANCER-VACCINES OR CANCERS OR CASE-REPORTS-IN-ONCOLOGICAL-MEDICINE OR CASE-REPORTS-IN-ONCOLOGY OR CELL-MOLECULAR-BIOLOGY-OF-PROSTATE-CANCER-UPDATES-INSIGHTS-AND-NEW-FRONTIERS OR CELLULAR-NUTRIENT-UTILISATION-AND-CANCER OR CHEMO-FOG-CANCER-CHEMOTHERAPY-RELATED-COGNITIVE-IMPARIMENT OR CHINESE-CLINICAL-ONCOLOGY OR CIRCULATING-TUMOUR-CELLS-IN-BREAST-CANCER-METASTATIC-DISEASE OR CLINICAL-ADVANCES-IN-HEMATOLOGY-ONCOLOGY OR CLINICAL-AND-TRANSLATIONAL-RADIATION-ONCOLOGY OR CLINICAL-CANCER-INVESTIGATION-JOURNAL OR CLINICAL-CANCER-PREVENTION OR CLINICAL-MEDICINE-INSIGHTS-ONCOLOGY OR CLINICAL-ONCOLOGY-IN-ADOLESCENTS-AND-YOUNG-ADULTS OR COLORECTAL-CANCER OR CONTROVERSIES-IN-TREATMENT-OF-LUNG- CANCER OR CONVERGENT-SCIENCE-PHYSICAL-ONCOLOGY OR CURRENT-BREAST-CANCER-REPORTS OR CURRENT-CANCER-THERAPY-REVIEWS OR CURRENT-COLORECTAL-CANCER-REPORTS OR DROSOPHILA-MODEL-IN-CANCER OR ECANCERMEDICALSCIENCE OR EMERGING-CONCEPTS-TARGETING-IMMUNE-CHECKPOINTS-IN-CANCER-AND-AUTOIMMUNITY OR EPIGENETICS-AND- CANCER-PT-A OR EPIGENETICS-AND-CANCER-PT-B OR EUROPEAN-UROLOGY-ONCOLOGY OR EXOSOMES-STEM-CELLS-AND-MICRORNA-AGING-CANCER-AND-AGE-RELATED-DISORDERS OR EXPERIMENTAL-HEMATOLGY-ONCOLOGY OR EXPERIMENTAL-HEMATOLOGY-ONCOLOGY OR FRONTIERS-IN-ONCOLOGY OR FRONTIERS-OF-RADIATION-THERAPY-AND-ONCOLOGY OR GACETA-MEXICANA-DE-ONCOLOGIA OR GYNECOLOGIC-ONCOLOGY-REPORTS OR HEPATIC-ONCOLOGY OR HETEROGENEITY-OF-CANCER-METABOLISM OR HORMONES-AND-BREAST-CANCER OR HSF1-AND-MOLECULAR-CHAPERONES-IN-BIOLOGY-AND-CANCER OR HUMAN-CELL-TRANSFORMATION-ADVANCES-IN-CELL-MODELS-FOR-THE-STUDY-OF-CANCER-AND-AGING OR HYPOXIA-AND-CANCER-METASTASIS OR IMPACT-OF-GENETIC-TARGETS-ON-CANCER-THERAPY OR IMPROVING-OUTCOMES-FOR-BREAST-CANCER-SURVIVORS-PERSPECTIVES-ON-RESEARCH-CHALLENGES-AND-OPPORTUNITIES OR IMRT-IGRT-SBRT-ADVANCES-IN-THE-TREATMENT-PLANNING-AND-DELIVERY-OF-RADIOTHERAPY OR INDIAN-JOURNAL-OF-GYNECOLOGIC-ONCOLOGY OR INDIAN-JOURNAL-OF-MEDICAL-AND-PAEDIATRIC-ONCOLOGY OR INDIAN-JOURNAL-OF-SURGICAL-ONCOLOGY OR INTERNATIONAL-CANCER-CONFERENCE-JOURNAL OR INTERNATIONAL-JOURNAL-OF-BREAST-CANCER OR INTERNATIONAL-JOURNAL-OF-CANCER-MANAGEMENT OR INTERNATIONAL-JOURNAL-OF-ENDOCRINE-ONCOLOGY OR INTERNATIONAL-JOURNAL-OF-SURGERY-ONCOLOGY OR INTERNATIONAL-JOURNAL-OF-SURGICAL-ONCOLOGY OR IRANIAN-JOURNAL-OF-CANCER-PREVENTION OR IRANIAN-JOURNAL-OF-PEDIATRIC-HEMATOLOGY-AND-ONCOLOGY OR JAMA-ONCOLOGY OR JCO-CLINICAL- CANCER-INFORMATICS OR JCO-GLOBAL-ONCOLOGY OR JCO-ONCOLOGY-PRACTICE OR JCO-PRECISION-ONCOLOGY OR JMIR-CANCER OR JNCI-CANCER-SPECTRUM OR JOURNAL-FOR-IMMUNOTHERAPY-OF-CANCER OR JOURNAL-OF-CANCER-EPIDEMIOLOGY OR JOURNAL-OF-CANCER-POLICY OR JOURNAL-OF-CANCER-PREVENTION OR JOURNAL-OF-COMMUNITY-AND-SUPPORTIVE-ONCOLOGY OR JOURNAL-OF-ENVIRONMENTAL-SCIENCE-AND-HEALTH-PART-C-ENVIRONMENTAL-CARCINOGENESIS-ECOTOXICOLOGY-REVIEWS OR JOURNAL-OF-EXPERIMENTAL-THERAPEUTICS-AND-ONCOLOGY OR JOURNAL-OF-GASTRIC-CANCER OR JOURNAL-OF-GASTROINTESTINAL-CANCER OR JOURNAL-OF-GASTROINTESTINAL-ONCOLOGY OR JOURNAL-OF-GLOBAL-ONCOLOGY OR JOURNAL-OF-HEPATOCELLULAR-CARCINOMA OR JOURNAL-OF-KIDNEY-CANCER-AND-VHL OR JOURNAL-OF-ONCOLOGY OR JOURNAL-OF-ONCOLOGY-PHARMACY-PRACTICE OR JOURNAL-OF-ONCOLOGY-PRACTICE OR JOURNAL-OF-PANCREATIC-CANCER OR JOURNAL-OF-RADIATION-ONCOLOGY OR JOURNAL-OF-RADIOTHERAPY-IN-PRACTICE OR JOURNAL-OF-SKIN-CANCER OR JOURNAL-OF-THE-EGYPTIAN-NATIONAL-CANCER-INSTITUTE OR LIVER-CANCER OR LONG-AND-SHORT-NONCODING-RNAS-IN-CANCER-BIOLOGY OR LUNG-CANCER-AND-AUTOIMMUNE-DISORDERS OR LUNG-CANCER-AND-PERSONALISED-MEDICINE-NOVEL-THERAPIES-AND-CLINICAL-MANAGEMENT OR LUNG-CANCER-MANAGEMENT OR LUNG-CANCER-TARGETS-AND-THERAPY OR MEMO-MAGAZINE-OF-EUROPEAN-MEDICAL-ONCOLOGY OR METALLOTHIONEINS-IN-NORMAL-AND-CANCER-CELLS OR MHC-CLASS-I-LOSS-AND-CANCER-IMMUNE-ESCAPE OR MICRORNA-CANCER-REGULATION-ADVANCED-CONCEPTS-BIOINFORMATICS-AND-SYSTEMS-BIOLOGY-TOOLS OR MIDDLE-EAST-JOURNAL-OF-CANCER OR MIRNAS-IN-AGING-AND-CANCER OR MOLECULAR-AND-CELLULAR-CHANGES-IN-THE-CANCER-CELL OR MOLECULAR-AND-CLINICAL-ONCOLOGY OR MOLECULAR-BIOLOGY- OF-CANCER-TRANSLATION-TO-THE-CLINIC OR MOLECULAR-CELLULAR-ONCOLOGY OR MOLECULAR-DIAGNOSTIC-IMAGING-IN-PROSTATE-CANCER-CLINICAL-APPLICATIONS-AND-TREATMENT-STRATEGIES OR MOLECULAR-GENETICS-OF-ENDOMETRIAL-CARCINOMA OR NANOMEDICINE-CANCER-DIABETES-AND-CARDIOVASCULAR-CENTRAL-NERVOUS-SYSTEM-PULMONARY-AND-INFLAMMATORY-DISEASES OR NASOPHARYNGEAL-CARCINOMA-KEYS-FOR-TRANSLATIONAL-MEDICINE-AND-BIOLOGY OR NATURAL-PRODUCTS-IN-CANCER-PREVENTION-AND-THERAPY OR NEURO-ONCOLOGY-PRACTICE OR NON-CODING-RNAS-IN-COLORECTAL-CANCER OR NOTCH-SIGNALING-IN-EMBRYOLOGY-AND-CANCER OR NOVEL-BIOMARKERS-IN-THE-CONTINUUM-

OF-BREAST-CANCER OR NPJ-BREAST-CANCER OR NPJ-PRECISION-ONCOLOGY OR NUTRITION-AND-PHYSICAL-ACTIVITY-IN-AGING-OBESITY-AND-CANCER OR OBESITY-FATTY-LIVER-AND-LIVER-CANCER OR OCULAR-ONCOLOGY-AND-PATHOLOGY OR ONCOLOGY-AND-THERAPY OR ONCOLOGY-IN-CLINICAL-PRACTICE OR ONCOLOGY-REVIEWS OR ONKOLOGIJA OR PERSONALISED-MEDICINE-LESSONS-FROM-NEURODEGENERATION-TO-CANCER OR POLYPLOIDISATION-AND-CANCER OR PRACTICAL-RADIATION-ONCOLOGY OR PROGRESS-IN-CANCER-IMMUNOTHERAPY OR PROMININ-1-CD133-NEW-INSIGHTS-ON-STEM-CANCER-STEM-CELL-BIOLOGY OR PROSTATE-CANCER OR PROSTATE-CANCER-CELLULAR-AND-GENETIC-MECHANISMS-OF-DISEASE- DEVELOPMENT-AND-PROGRESSION-2ND-EDITION OR RADIATION-ONCOLOGY-JOURNAL OR RECENT-RESULTS-IN-CANCER-RESEARCH OR REGULATION-OF-CANCER-IMMUNE-CHECKPOINTS-MOLECULAR-AND-CELLULAR-MECHANISMS-AND-THERAPY OR REHABILITATION-ONCOLOGY OR REPORTS-OF-PRACTICAL-ONCOLOGY-AND-RADIOTHERAPY OR REPRODUCTIVE-HEALTH-AND-CANCER-IN-ADOLESCENTS-AND-YOUNG-ADULTS OR RESPIRATORY-CARCINOGENESIS OR REVISTA-COLOMBIANA-DE-CANCEROLOGIA OR REVUE-D-ONCOLOGIE-HEMATOLOGIE-PEDIATRIQUE OR ROLE-OF-BIOACTIVE-LIPIDS-IN-CANCER-INFLAMMATION-AND-RELATED-DISEASES OR RUNX-PROTEINS-IN-DEVELOPMENT-AND-CANCER OR SEMINARS-IN-ONCOLOGY-NURSING OR SOUTH-ASIAN-JOURNAL-OF-CANCER OR SOUTHERN-AFRICAN-JOURNAL-OF-GYNAECOLOGICAL-ONCOLOGY OR STEM-CELLS-HETEROGENEITY-IN-CANCER OR STEM-CELLS-PRE-NEOPLASIA-AND-EARLY-CANCER-OF-THE-UPPER-GASTROINTESTINAL-TRACT OR TARGETED-THERAPY-OF-COLORECTAL-CANCER-SUBTYPES OR TOWARD-PERSONALISED-MEDICINE-FOR-CANCER OR TRANSLATIONAL-CANCER-RESEARCH OR TRANSLATIONAL-GASTROINTESTINAL-CANCER OR TRANSLATIONAL-LUNG-CANCER-RESEARCH OR TRANSLATIONAL-RESEARCH-AND-ONCO-OMICS-APPLICATIONS-IN-THE-ERA-OF-CANCER-PERSONAL-GENOMICS OR TRANSLATIONAL-RESEARCH-IN-BREAST-CANCER-BIOMARKER-DIAGNOSIS-TARGETED-THERAPIES-AND-APPROACHES-TO-PRECISION-MEDICINE OR TRENDS-IN-CANCER OR TURK-ONKOLOGI-DERGISI-TURKISH-JOURNAL-OF-ONCOLOGY OR TURK-ONKOLOJI-DERGISI-TURKISH-JOURNAL-OF-ONCOLOGY OR UROONKOLOJI-BULTENI-BULLETIN-OF-UROONCOLOGY OR VIRUSES-GENES-AND-CANCER OR WORLD-CANCER-RESEARCH-JOURNAL OR WORLD-JOURNAL-OF-CLINICAL-ONCOLOGY OR WORLD-JOURNAL- OF-GASTROINTESTINAL-ONCOLOGY OR WORLD-JOURNAL-OF-ONCOLOGY)

TI=((ANTITUMOUR* NOT NECROSIS) OR (HPV* NOT PARVO*) OR (IRRADIATION AND FRACTIONATED) OR (MYC NOT (C OR N)) OR (PML AND (APOPTO* OR GENE OR NUCLEAR OR NUCLEUS OR PROTEIN* OR RAR* OR UBIQUITIN)) OR (TOPOISOMERASE AND INHIBITOR) OR (TUMO*R* NOT NECROSIS) OR 5T4 OR ADENOCARCINOMA OR ADENOCARCINOMAS OR ADENOMA OR ADENOMAS OR ADENOSARCOMAS OR ADENOSARCOMA OR ADRIAMYCIN OR AGR3 OR AKAP13 OR ALEMTUZUMAB OR ALEX2 OR ALTRETAMINE OR AMELOBLASTOMA OR AMELOBLASTOMAS OR AMIFOSTINE OR AML OR ANASTROZOLE OR ANGIOSARCOMA OR ANGIOSARCOMAS OR ANTICANCER* OR ANTICARCINO* OR ANTILEUKEMIC OR ANTIMELANOMA OR ANTIMYELOMA OR ANTINEOPLAS* OR ANTIPROLIF* OR ANTITUMO*R OR ARIMIDEX* OR ARMCX1 OR AROMATASE OR ASTROCYTOMA OR ASTROCYTOMAS OR AZACITIDINE OR B-8801 OR BCAR1 OR BCL2 OR BCL2 OR BCR-ABL OR BCR/ABL OR BICALUTAMIDE OR BIN2 OR BIOREDUCTIVE OR BLEOMYCIN OR BORTEZOMIB OR BRAF OR BRAP1 OR BRCA OR BRCA1 OR BRCA2 OR BRCC3 OR BRACHYTHERAPY OR BRI3BP OR BRMS1 OR BRYOSTATIN* OR BUSULFAN OR C2ORF40 OR CAELYX* OR CAGE1 OR CAGE-1 OR (CANCER* NOT (CRAB OR LYNX-CANCER OR TROPIC)) OR CAPECITABINE OR CARBOGEN OR CARBOPLATIN OR (CARCINO* NOT (CARCINOSCORPUS OR CARCINONEMERT*)) OR CARMUSTINE OR CDKN2A OR CDX2 OR CEA OR CEP290 OR CERVICAL SMEAR OR CETUXIMAB OR CHEMOPREVENT* OR CHEMORADIOTHERAPY OR CHEMOSENSITIV* OR (CHEMOTHERAP* NOT (MALARIA OR TB OR TUBERCULOSIS)) OR CHLORAMBUCIL OR CHOLANGIOCARCINOMA OR CHONDROSARCOMA OR CHORIOCARCINOMA OR CIN OR CISPLATIN OR CLADRIBINE OR CLL OR CML OR COMBRETASTATIN OR CRANIOPHARYNGIOMA OR CT45-1 OR CT47 OR CYCLOPHOSPHAMIDE OR CYSTADENOCARCINOMA OR CYSTADENOMA OR CYTARABINE OR CYTOSINE-ARABINOSIDE OR DACARBAZINE OR DASATINIB OR DAUNORUBICIN OR DBC1 OR DDX53 OR DECITABINE OR DERMATOFIBROSARCOMA OR DOCETAXEL OR DOXORUBICIN* OR DU-PAN-2 OR DYSGERMINOMA OR DYSGERMINOMAS OR EBAG9 OR ECRG OR EEF1A1 OR ELAC2 OR EORTC OR EPENDYMOMA OR EPIRUBICIN OR ERBB* OR ERLOTINIB OR ESTRAMUSTINE OR ETOPOSIDE* OR ETV2 OR EXEMESTANE OR FIBROMA OR FIBROSARCOMA OR FLI1 OR FLOXURIDINE OR FLUOROURACIL OR FOS OR FULVESTRANT OR GA50 OR GANGLIOGLIOMA OR GANGLIOGLIOMAS OR GANGLIONEUROBLASTOMA OR GEFITINIB OR GEMCITABINE OR GEMTUZUMAB OR GERMINOMA OR GLEASON OR GLEVEC* OR GLIOBLASTOMA OR GLIOMA OR GLIOSARCOMA OR GLIVEC OR GOSERELIN OR HCCR1 OR HEMANGIOBLASTOMA OR HEMANGIOENDOTHELIOMA OR HEMANGIOSARCOMA OR HEPATOBLASTOMA OR HEPATOCARCINO* OR HEPATOMA OR HER2 OR HERCEPTIN* OR HISTIOCYTOMA OR HODGKIN DISEASE OR HODGKINS OR HRPT2 OR HYPERNEPHROMA

OR IBRITUMOMAB-TIUXETAN OR IDARUBICIN OR IFOS*AMIDE* OR IMATINIB OR IMRT OR INSULINOMA OR INTRATUMOUR* OR IODINE-131-ANTI-B1-ANTIBODY OR IPILIMUMAB OR IRESSA OR IRINOTECAN OR IXABEPILONE OR JUN OR L514S OR L552S OR LAPATINIB OR LCAP OR LEIOMYOMA OR LEIOMYOSARCOMA OR LENALIDOMIDE OR LETMD1 OR LETROZOLE OR LEUKAEM* OR LEUKEM* OR LI FRAUMENI OR LIPOSARCOMA OR LOMUSTINE OR LY2K OR LYMPHOBLASTIC OR LYMPHOMA* OR LYMPHOPROLIFERATIVE OR MACC1 OR MALIGNANC* OR MALIGNANT OR MAMMOGRA* OR MAP3K8 OR MASTECTOM* OR MEDULLOBLASTOMA OR MELANOMA OR MELPHALAN* OR MENINGIOMA OR MERCAPTOPURINE OR MESOTHELIOMA OR METASTAS* OR METASTAT* OR METHYLGUANINE OR MITOMYCIN OR MITOXANTRONE OR MLH1 OR MLL OR MSH OR MSH2 OR MUC1 OR MYB OR MYELODYSPLAS* OR MYELOID OR MYELOMA* OR MYELOPROLIFERATIVE OR MYXOFRIBOSARCOMA OR NEOPLAS* OR NEPHROBLASTOMA OR NEPHROMA OR NEURINOMA OR NEUROBLASTOMA OR NEUROFIBROSARCOMA OR NEUROMA OR NHL OR NSCLC OR NUP98 OR OLIGOASTROCYTOMA OR OLIGODENDROGLIOMA OR ONCOGEN* OR ONCOLOG* OR ONCOLYTIC OR ONCOPROTEIN OR OSTEOSARCOMA OR OXALIPLATIN OR P12INK4A OR PANITUMAB OR PAP-SMEAR OR PAPILLOMA OR PAX3 OR PBOV1 OR PEGASPARGASE OR PEMETREXED OR PENTOSTATIN OR PEUTZ OR PHEOCHROMOCYTOMA OR PHTOTODYNAMIC-THERAP* OR PLASMACYTOMA OR PML/RAR* OR PMS1 OR POLYCYTHEMIA-RUBRA OR PPHLN1 OR PROCARBAZINE OR PROSTATECTOMY OR PROTOONCOGEN* OR PROSTATE-SPECIFIC-ANTIGEN OR PTEN OR RAD51 OR RADIATION-THERAPY OR RADIOSENSI* OR RADIOSURGERY OR RADIOTHERAP* OR RALTITREXED OR RB1 OR RCVRN OR RET OR RETINOBLASTOMA OR RHABDOMYOSARCOMA OR RHOBTB2 OR SARCOMA OR SCHWANNOMA OR SDCCAG OR SEMINOMA OR SFXN4 OR SKCG-1 OR SLC35C2 OR SNCG OR SORAFENIB OR SPANXC OR SRC OR STEAP2 OR SUNITINIB OR TAMOXIFEN OR TARCEVA OR TAXOL OR TAXOTERE OR TBC1D3 OR TCCSG OR TEMODAL OR TEMOZOL*MIDE OR TEMSIROLIMUS OR TENIPOSIDE OR TERATOMA OR TFF1 OR THIOGUANINE OR THIOTEPA OR THYMOMA OR TOMOTHERAPY OR TOMUDEX OR TOPOTECAN OR TP53 OR TRASTUZUMAB OR TREOSULFAN OR TROVAX OR TSC1 OR UOEH-LC-1 OR VCRP PROTOCOL OR VINBLASTINE OR VINCRISTINE OR VINORELBINE OR VWA5A OR WALDENSTROM* OR XAGE1A OR XERODERMA-PIGMENTOSUM OR ZOLEDRONIC-ACID OR ABIRATERONE OR ANTHRACYCLINE OR ANTHRACYCLINES OR ANTILEUKEMIA OR AXITINIB OR BLINATUMOMAB OR BOSUTINIB OR BRENTUXIMAB OR CARFILZOMIB OR CATUMAXOMAB OR CEDIRANIB OR CERITINIB OR CHEMORADIATION OR CHORDOMA OR CRIZOTINIB OR CYSTECTOMY OR DCIS OR DINACICLIB OR DOVITINIB OR ENZALUTAMIDE OR ERIBULIN OR ESOPHAGECTOMY OR ESTHESIONEUROBLASTOMA OR FUNGOIDES OR (GIST AND GASTR*) OR HCC OR HNSCC OR IBRUTINIB OR IDELALISIB OR LIPOBLASTOMA OR LYMPHADENECTOMY OR LYNCH-SYNDROME OR NILOTINIB OR OESOPHAGECTOMY OR OSTEOCHONDROMA OR OSTEOCHONDROMAS OR PACLITAXEL OR PANCREATICODUODENECTOMY OR PANCREATOBLASTOMA OR PANCREATODUODENECTOMY OR PANITUMUMAB OR PARANEOPLASTIC OR PAZOPANIB OR POSTMASTECTOMY OR PROTON-BEAM-THERAPY OR PSEUDOMYXOMA OR PSEUDOMYXOMAS OR REGORAFENIB OR SBRT OR TRAMETINIB OR VEMURAFENIB OR VISMODEGIB OR VMAT)

### Appendix B

**Table:** Summary table of all included studies.

Reference Year	Author	Study	Funding	Zambian first authorship	Zambian last authorship	Research design	Sample size	Data type	Data period	Cancer care pathway	Cancer type	Three-delay framework
2012	White *et al* [31]	Motivations and experiences of women who accessed see and treat cervical cancer prevention services in Zambia	National Cancer Institute	No	No	Prospective, observational	81	Primary	2009–2010	Diagnostics	Cervical	Delay in reaching care
2012	White *et al* [32]	Worse than HIV' or 'not as serious as other diseases'? Conceptualization of cervical cancer among newly screened women in Zambia	National Cancer Institute	No	No	Prospective, observational	60	Primary	2009–2010	Diagnostics	Cervical	Delay in seeking care
2013	Kayamba *et al* [57]	Gastric adenocarcinoma in Zambia: a case-control study of HIV, lifestyle risk factors and biomarkers of pathogenesis	National Institute of Health (NIH); American Relief and Recovery Act; Fogarty International Centre	Yes	No	Retrospective, observational	146	Primary	2010–2012	Aetiology	Gastric	N/A
2013	Asombang *et al* [58]	Gastric cancer in Zambian adults: a prospective case-control study that assessed dietary intake and antioxidant status by using urinary isoprostane excretion	National Institute of Health (NIH); American Relief and Recovery Act; Fogarty International Centre	No	No	Prospective, observational	140	Primary	2010–2012	Aetiology	Gastric	N/A
2013	Mwanahamuntu *et al* [89]	Utilization of cervical cancer screening services and trends in screening positivity rates in a 'Screen-And-Treat' program integrated with HIV/AIDS care in Zambia	Zambian Ministry of Health; U.S. President's Emergency Plan for AIDS Relief (PEPFAR) program through the US Centers for Disease Control and Prevention (CDC); National Institutes of Health (NIH)/ Fogarty International Center	Yes	Yes	Retrospective, observational	56,247	Secondary	2006–2011	Epidemiology	Cervical	N/A
2013	Kapambwe *et al* [33]	Innovative approaches to promoting cervical health and raising cervical cancer awareness by use of existing cultural structures in resource-limited countries: experiences with traditional marriage counseling in Zambia	N/A	Yes	Yes	Prospective, observational	70	Primary	2012	Diagnostics	Cervical	Delay in seeking care
2014	Bateman *et al* [34]	Clinical performance of digital cervicography and cytology for cervical cancer screening in HIV-infected women in Lusaka, Zambia	U.S. National Cancer Institute; Fogarty International Center (FIC); WHO; World Bank	No	No	Retrospective, observational	303	Primary	2008–2011	Diagnostics	Cervical	Delay in receiving care
2014	Slone *et al* [77]	Pediatric malignancies, treatment outcomes and abandonment of pediatric cancer treatment in Zambia	National Institutes of Health (NIH); Cancer Research UK; Eunice Kennedy Shriver National Institute of Child Health & human Development	No	No	Retrospective, observational	162	Secondary	2008–2011	Treatment	Other- paediatric	N/A
2014	Maree *et al* [35]	Zambian women's experiences and understanding of cervical cancer A qualitative study	N/A	No	No	Prospective, observational	21	Primary	Not specified	Diagnostics	Cervical	Delay in seeking care
2014	Rohner *et al* [90]	Incidence rate of Kaposi sarcoma in HIV-infected patients on antiretroviral therapy in Southern Africa: a prospective multicohort study	National Institute of Allergy and Infectious Disease	No	No	Retrospective, observational	173,245	Secondary	2004–2010	Epidemiology	Kaposi Sarcoma	N/A
2015	Mulele et al [26]	Observed and expected incidence of cervical cancer in Lusaka and the Southern and Western Provinces of Zambia, 2007 to 2012	U.S. National Cancer Institute; Fogarty International Center (FIC)	Yes	No	Retrospective, observational	4,498	Secondary	2007–2012	Epidemiology	Cervical	N/A
2015	Bateman *et al* [91]	The burden of cervical pre-cancer and cancer in HIV positive women in Zambia: a modeling study	U.S. National Cancer Institute; Fogarty International Center (FIC); National Institute of Allergy and Infectious Disease	No	No	Retrospective, observational	309	Primary	2008–2011	Epidemiology	Cervical	N/A
2015	Chamot *et al* [36]	Preference for Human Papillomavirus-Based cervical cancer screening: results of a choice-based conjoint study in Zambia	National Cancer Institute	No	Yes	Prospective, observational	238	Primary	Not specified	Diagnostics	Cervical	Delay in reaching care
2015	Kayamba *et al* [59]	HIV infection and domestic smoke exposure, but not human papillomavirus, are risk factors for esophageal squamous cell carcinoma in Zambia: a case-control study	Fogarty International Center (FIC); National Institutes of Health (NIH); National Heart, Blood, and Lung Institute; National Institute of Mental Health	Yes	No	Retrospective, observational	222	Primary	2013–2014	Aetiology	Oesophageal	N/A
2015	Kapambwe *et al* [60]	Age distribution and determinants of invasive cervical cancer in a screen-and-treat program integrated With HIV/AIDS care in Zambia	President's Emergency Plan for AIDS Relief (PEPFAR); National Institutes of Health (NIH); Fogarty International Center (FIC)	Yes	Yes	Retrospective, observational	48,626	Secondary	2006–2010	Aetiology	Cervical	N/A
2015	Bateman *et al* [61]	Identification of Human Papilloma Viruses from formalin-fixed, paraffin-embedded pre-cancer and invasive cervical cancer specimens in Zambia: a cross-sectional study	Program in Global Oncology of the UNC Lineberger Cancer Center; Fogarty International Center (FIC); National Cancer Institute (NCI); Nationa Institute of Health (NIH)	No	No	Cross-sectional, observational	27	Primary	2007–2012	Aetiology	Cervical	N/A
2015	Parham *et al* [37]	Population-level scale-up of cervical cancer prevention services in a low-resource setting: development, implementation, and evaluation of the cervical cancer prevention program in Zambia	Zambian Ministry of Health; Ministry of Community Development, Mother and Child Health; U.S. President's Emergency Plan for AIDS Relief (PEPFAR); Fogarty International Center (FIC); National Institutes of Health (NIH)	Yes	No	Retrospective, observational	102,942	Secondary	2006–2013	Diagnostics	Cervical	Delay in reaching care; delay in receiving care
2016	Mdletshe *et al* [78]	Acute toxicity in cervical cancer HIV-positive versus HIV-negative patients treated by radical chemo-radiation in Zambia	N/A	No	Yes	Prospective, observational	120	Primary	2014–2015	Treatment- radiotherapy, chemotherapy	Cervical	N/A
2016	Kayamba *et al* [62]	Serological response to Epstein-Barr virus early antigen is associated with gastric cancer and human immunodeficiency virus infection in Zambian adults: a case-control study	Wellcome Trust	Yes	No	Retrospective, observational	147	Primary	2010–2012	Aetiology	Gastric	N/A
2016	Chibweseha *et al* [38]	Clinical performance validation of four point-of-care cervical cancer screening tests in HIV-infected women in Zambia	National Cancer Institute; Fogarty International Center; Fulbright-Fogarty Fellowship Award; Eunice Kennedy Shriver National Institute of Child Health & Human Development	No	Yes	Retrospective, observational	200	Primary	2015	Diagnostics	Cervical	Delay in receiving care
2016	Asombang *et al* [63]	Esophageal squamous cell cancer in a highly endemic region	American Relief and Recovery Act; Siteman Comprehensive Cancer Center; National Cancer Institute (NCI); Fogarty International Center (FIC); National Institutes of Health (NIH)	No	No	Retrospective, observational	72	Primary	2010–2012	Aetiology	Oesophageal	N/A
2017	Tembo *et al* [64]	Detection of Human Herpes Virus 8 in Kaposi's sarcoma tissues at the University Teaching Hospital, Lusaka, Zambia	University of Zambia Staff Development Office; Medical Education Partnership Initiative	Yes	Yes	Retrospective, observational	84	Primary	2013–2014	Aetiology	Kaposi Sarcoma	N/A
2017	Maree *et al* [96]	Caring for patients with advanced breast cancer: the experiences of Zambian nurses	N/A	No	Yes	Prospective, observational	17	Primary	2014	Palliative Care	Breast	N/A
2017	Hamoonga *et al* [39]	Higher educational attainment associated with reduced likelihood of abnormal cervical lesions among Zambian women - a cross sectional study	Research Council of Norway through Centres of Excellence Scheme; Global Health and Vaccination Programme; Wellcome Trust; Department for International Development; Alliance for Accelerating Excellence in Science in Africa (DELTAS)	Yes	Yes	Retrospective, observational	14,294	Secondary	2013–2014	Diagnostics	Cervical	Delay in seeking care
2017	Pinder *et al* [40]	Leverage of an existing cervical cancer prevention service platform to initiate breast cancer control services in Zambia: experiences and early outcomes	Fogarty International Center (FIC); National Institutes of Health (NIH)	No	Yes	Prospective, observational	1,955	Secondary	2015–2016	Diagnostics, treatment	Breast	Delay in reaching care; delay in receiving care
2018	Kafita *et al* [65]	Evidence of EBV infection in lymphomas diagnosed in Lusaka, Zambia	OGAC; OAR; University of Zambia Staff Development Office; Fogarty International Center (FIC)	Yes	Yes	Retrospective, observational	150	Primary	2011–2014	Aetiology	Other- lymphoma	N/A
2018	Mtonga *et al* [79]	Therapeutic outcomes in AIDS-associated Kaposi's sarcoma patients on antiretroviral therapy treated with chemotherapy at two Tertiary Hospitals in Lusaka, Zambia	Fogarty International Center (FIC); National Institutes of Health (NIH)	Yes	Yes	Retrospective, observational	38	Primary	2015–2016	Treatment- chemotherapy	Kaposi Sarcoma	N/A
2018	Nyambe *et al* [41]	The impact of the social environment on Zambian cervical cancer prevention practices	VLIR-UOS	No	No	Retrospective, observational	61	Primary	2016	Prevention; diagnostics	Cervical	Delay in seeking care; delay in reaching care; delay in receiving care
2018	Walubita *et al* [42]	Challenges for health care providers, parents and patients who face a child hood cancer diagnosis in Zambia	N/A	Yes	Yes	Prospective, observational	22	Primary	2014	Diagnostics, treatment	Other- paediatric	Delay in seeking care; delay in reaching care; delay in receiving care; non-compliance to treatment
2018	Wigginton *et al* [43]	Death, contagion and shame: the potential of cancer survivors' advocacy in Zambia	Australian National Breast Cancer Foundation	No	No	Prospective, observational	13	Primary	2017	Diagnostics	Breast	Delay in seeking care
2018	Asombang *et al* [44]	Descriptive analysis of colorectal cancer in Zambia, Southern Africa using the National Cancer Disease Hospital Database	American College of Gastroenterology; North American International GI Training Grant Award	No	Yes	Retrospective, observational	377	Secondary	2007–2015	Diagnostics	Other- colorectal	Delay in seeking care
2018	Nyambe *et al* [45]	Differences in cervical cancer screening knowledge and practices by HIV status and geographic location: implication for program implementation in Zambia	US National Cancer Institute (NCI)	No	No	Prospective, observational	59	Primary	2017	Diagnostics	Cervical	Delay in seeking care
2018	Kayamba *et al* [46]	Presence of blood in gastric juice: a sensitive marker for gastric cancer screening in a poor resource setting	Fogarty International Center (FIC); National Institutes of Health (NIH)	Yes	No	Prospective, observational	276	Primary	2016–2017	Diagnostics	Gastric	Delay in receiving care
2018	McKenzie *et al* [6]	Drivers of advanced stage at breast cancer diagnosis in the multicountry ABC-DO study	Susan G Komen for the Cure Foundation; National Cancer Institute (NCI)	No	No	Prospective, observational	1,795	Primary	2014–2017	Diagnostics	Breast	Delay in seeking care
2019	Nyambe *et al* [47]	Using film to disseminate information on cervical cancer prevention in Lusaka: results from a small intervention study	N/A	No	No	Prospective, observational	81	Primary	2017	Diagnostics	Cervical	Delay in seeking care
2019	Sibulwa *et al* [80]	Every part of me has changed-shared lived experiences of adolescents living with cancer in Zambia	N/A	Yes	Yes	Prospective, observational	18	Primary	2018	Treatment, palliative care	Other- paediatric	N/A
2019	Hamoonga *et al* [63]	Vaginal douching in Zambia: a risk or benefit to women in the fight against cervical cancer: a retrospective cohort study	Fogarty International Center (FIC); National Institutes of Health (NIH)	Yes	Yes	Retrospective, observational	11,853	Secondary	2006–2014	Aetiology	Cervical	N/A
2019	Nyambe *et al* [48]	Knowledge, attitudes and practices of cervical cancer prevention among Zambian women and men	VLIR-UOS	No	No	Prospective, observational	600	Primary	2016	Prevention; diagnostics	Cervical	Delay in seeking care
2019	Utter *et al* [95]	Availability of palliative care services in Zambia: a nationwide provincial and tertiary hospital survey	Susan G. Komen Foundation; Fulbright-Fogarty Fellowship Award; Fogarty International Center (FIC); National Cancer Institute (NCI)	Yes	No	Cross-sectional, observational	11	Primary	2014–2015	Palliative Care	Other- not specified	N/A
2019	Kapambwe *et al* [49]	Partnering with traditional Chiefs to expand access to cervical cancer prevention services in rural Zambia	Fogarty International Center (FIC); National Institutes of Health (NIH)	Yes	Yes	Prospective, observational	8,399	Primary	2015–2016	Diagnostics	Cervical	Delay in seeking care
2019	Bing *et al* [81]	Using low-cost virtual reality simulation to build surgical capacity for cervical cancer treatment	UK Research and Innovation Global Challenges Research Fund (GCRF)]; Dedman College Interdisciplinary Institute; Center for Global Health Impact at Southern Methodist University	No	No	Prospective, observational	10	Primary	2017	Treatment- surgery	Cervical	N/A
2020	Kasochi *et al* [67]	Human epidermal growth factor receptor 2 overexpression in gastric and gastroesophageal junction adenocarcinoma in patients seen at the University Teaching Hospital, Lusaka, Zambia	U.S Civilian Research & Development Foundation (CRDF Global); Fogarty International Center (FIC); National Institutes of Health (NIH)	Yes	Yes	Retrospective, observational	57	Primary	2015–2018	Aetiology	Gastric	N/A
2020	Okuku *et al* [68]	Molecular detection of fusion oncogenes in zambian patients with acute lymphoblastic leukemia	N/A	Yes	Yes	Retrospective, observational	19	Primary	2015–2016	Aetiology	Other- leukaemia	N/A
2020	Trejo *et al* [69]	Effects of HIV status on non-metastatic cervical cancer progression among patients in Lusaka, Zambia	National Cancer Institute	No	No	Retrospective, observational	577	Secondary	2008–2012	Aetiology	Cervical	N/A
2020	Gift *et al* [50]	Assessment of knowledge, practice and attitude towards prostate cancer screening among male patients aged 40 years and above at Kitwe Teaching Hospital, Zambia	Zambian Ministry of Higher Education	Yes	Yes	Prospective, observational	200	Primary	2019	Diagnostics	Other- prostate	Delay in seeking care
2020	Kalubula *et al* [28]	Epidemiology of Kaposi's sarcoma in Zambia, 2007–2014	Institute of Urban Environment; University of Chinese Academy of Sciences (UCAS)	No	Yes	Retrospective, observational	2,521	Secondary	2007–2014	Epidemiology	Kaposi Sarcoma	N/A
2020	Vally *et al* [51]	Admitted AIDS-associated Kaposi sarcoma patients indications for admission and predictors of mortality	Fogarty International Center (FIC); National Institutes of Health (NIH)	Yes	Yes	Retrospective, observational	54	Primary	2019–2020	Diagnostics	Kaposi Sarcoma	Delay in receiving care
2020	Kayamba *et al* [70]	Molecular profiling of gastric cancer in a population with high HIV prevalence reveals a shift to MLH1 loss but not the EBV subtype	Fogarty International Center (FIC); National Institutes of Health (NIH)	Yes	No	Retrospective, observational	369	Primary	2016–2018	Aetiology	Gastric	N/A
2020	Songiso *et al* [52]	Minimizing delays in the breast cancer pathway by integrating breast specialty care services at the primary health care level in Zambia	Susan G. Komen Foundation; University of North Carolina at Chapel Hill; Zambian Ministry of Health; University of Washington	Yes	Yes	Prospective, observational	1,790	Primary	2018–2019	Diagnostics, treatment	Breast	Delay in seeking care; delay in reaching care; delay in receiving care
2020	Kayamba *et al* [71]	Biomass smoke exposure is associated with gastric cancer and probably mediated via oxidative stress and DNA damage: a case-control study	US Civilian Research and Development Foundation (CRDF Global); Fogarty International Center (FIC); National Institutes of Health (NIH)	Yes	No	Retrospective, observational	316	Primary	2016–2018	Aetiology	Gastric	N/A
2020	Stecklein *et al* [82]	Radiation sciences education in Africa: an assessment of current training practices and evaluation of a high-yield course in radiation biology and radiation physics	National Cancer Institute of the National Institutes of Health]; University of Texas MDA Cancer Center	No	Yes	Prospective, observational	36	Primary	2020	Treatment- radiotherapy	Other- not specified	N/A
2020	Pinder *et al* [83]	Thermal ablation versus cryotherapy or loop excision to treat women positive for cervical precancer on visual inspection with acetic acid test: pilot phase of a randomised controlled trial	National Cancer Institute	No	No	Randomised controlled trial	750	Primary	2017–2019	Treatment- surgery	Cervical	N/A
2020	McCormack *et al* [92]	Breast cancer survival and survival gap apportionment in sub-Saharan Africa (ABC-DO): a prospective cohort study	Susan G Komen; IARC; National Institute for Health	No	No	Retrospective, observational	2,313	Primary	2014–2017	Epidemiology	Breast	N/A
2021	Julius *et al* [72]	Clinical and pathologic presentation of primary ocular surface tumors among Zambians	National Institute of Health (NIH)	Yes	No	Retrospective, observational	265	Primary	2017–2019	Aetiology	Other- ocular	N/A
2021	Pattee *et al* [84]	The use a virtual interactive system to enhance gynecologic oncology multi-disciplinary care in Zambia	Nancy Sanders Memorial Faculty Research Abroad Grant from the Global Engagement Office of West Virginia University Health Sciences	No	Yes	Prospective, observational	130	Primary	2021	Treatment	Cervical	N/A
2021	Asombang *et al* [93]	Descriptive analysis of esophageal cancer in Zambia using the cancer disease hospital database: young age, late stage at presentation	N/A	No	Yes	Retrospective, observational	278	Secondary	2007–2018	Epidemiology	Oesophageal	N/A
2021	Mumba *et al* [4]	Cervical cancer diagnosis and treatment delays in the developing world: evidence from a hospital-based study in Zambia	N/A	Yes	No	Retrospective, observational	2,121	Primary	2014–2018	Diagnostics	Cervical	Delay in receiving care
2021	Pry *et al* [53]	Cervical cancer screening outcomes in Zambia, 2010–19: a cohort study	US President's Emergency Plan for AIDS Relief	No	Yes	Retrospective, observational	183,165	Secondary	2010–2019	Diagnostics	Cervical	Delay in seeking care; delay in reaching care
2021	Kalubula *et al* [29]	Epidemiology of cancers in Zambia: a significant variation in cancer incidence and prevalence across the nation	University of Chinese Academy of Sciences (UCAS)	No	No	Retrospective, observational	21,512	Secondary	2007–2014	Epidemiology	Other- not specified	N/A
2021	Hayumbu *et al* [94]	Cervical cancer and precancerous cervical lesions detected using visual inspection with acetic acid at Livingstone Teaching Hospital	N/A	Yes	Yes	Retrospective, observational	329	Primary	2019–2020	Epidemiology	Cervical	N/A
2021	Bing *et al* [85]	User experience with low-cost virtual reality cancer surgery simulation in an African setting	UK Research and Innovation; Dedman College Interdisciplinary Institute at Southern Methodist University; Center for Global Health Impact at Southern Methodist University; Wellcome Trust	No	Yes	Prospective, observational	11	Primary	2019	Treatment- surgery	Cervical	N/A
2021	Galukande *et al* [104]	Maternally orphaned children and intergenerational concerns associated with breast cancer deaths among women in Sub-Saharan Africa	Susan G. Komen; National Cancer Institute; IARC	No	No	Retrospective, observational	795	Secondary	2014–2019	N/A	Breast	N/A
2021	Togawa *et al* [54]	Geospatial barriers to healthcare access for breast cancer diagnosis in sub-Saharan African settings: the ABC-DO cohort study	Centre International de Recherche sur le Cancer; Susan G. Komen	No	No	Prospective, observational	1,941	Primary	2014–2017	Diagnostics	Breast	Delay in reaching care
2022	Trejo *et al* [55]	Effects of HIV infection on metastatic cervical cancer and age at diagnosis among patients in Lusaka, Zambia	Cancer Epidemiology Education in Special Populations (CEESP) Program; National Cancer Institute	No	Yes	Retrospective, observational	1,593	Secondary	Not specified	Diagnostics	Cervical	Delay in seeking care
2022	Diao *et al* [105]	Perspectives of Zambian Clinical Oncology Trainees in the MDA and Zambia virtual clinical research training program (MOZART)	National Cancer Institute, National Institutes of Health; Derek Harwood-Nash International Education Scholar Grant through the Radiological Society of North America (RSNA); MDA Cancer Center Department of Radiation Oncology Strategic Initiatives (ROSI) grant	No	No	Retrospective, observational	14	Primary	2021	Research	Other- not specified	N/A
2022	Zyambo *et al* [73]	Evaluation of the association between gastric cancer and plasma selenium in Zambian adults: a case-control study	U.S Civilian Research and Development Foundation (CRDF Global); Fogarty International Center (FIC); National Institutes of Health (NIH)	Yes	Yes	Retrospective, observational	159	Primary	2019–2020	Aetiology	Gastric	N/A
2022	Julius *et al* [74]	Epstein-Barr Virus, But Not Human Papillomavirus, Is Associated With Preinvasive and Invasive Ocular Surface Squamous Neoplasias in Zambian Patients	National Institutes of Health; Zambia AIDS Malignancies Diagnosis and Pathogenesis Program (ZAMDAPP); PCA from the UNMC Cancer Center	Yes	No	Prospective, observational	243	Primary	2017–2020	Aetiology	Other- ocular	N/A
2022	Maate *et al* [75]	High-risk Human Papillomavirus-associated vulvar neoplasia among women living with human immunodeficiency virus in Zambia	University of North Carolina at Chapel Hill; Johns Hopkins University; Morehouse School of Medicine; Tulane University Fogarty Global Health Fellowship Program	Yes	Yes	Retrospective, observational	53	Primary	2017–2018	Aetiology	Other- vulva	N/A
2022	Mwanahamuntu *et al* [86]	The use of thermal ablation in diverse cervical cancer screen-and-treat service platforms in Zambia	US National Institute of Health; University of North Carolina; University of Washington	Yes	Yes	Retrospective, observational	2,123	Secondary	2012–2020	Treatment- surgery	Cervical	N/A
2022	Riebensahm *et al* [27]	Screening for hepatocellular carcinoma among adults with HIV/HBV co-infection in Zambia: a pilot study	National Institute of Allergy and Infectious Disease; Fogarty International Center at the National Institutes of Health (NIH); Swiss Cancer League; Swiss National Science Foundation	No	No	Prospective, observational	279	Primary	2015–2020	Diagnostics	Other-liver	Delay in reaching care
2022	Kayamba *et al* [56]	Association between oesophageal cancer and biomass smoke exposure: a case-control study	N/A	Yes	No	Retrospective, observational	366	Primary	2018–2021	Aetiology	Oesophageal	N/A
2022	Hicks *et al* [87]	The evolution of a novel approach to building surgical capacity for cervical cancer in Africa	N/A	No	Yes	Prospective, observational	N/A	Primary	2012–2019	Treatment- surgery	Cervical	N/A
2022	Sowerbutts *et al* [76]	A qualitative exploration of nutrition screening, assessment and oral support used in patients undergoing cancer surgery in LMICs	Medical Research Council; National Institute for Health Research (NIHR) using UK aid from the UK Government [NIHR]; Cancer Research UK Career Development Fellowship	No	No	Retrospective, observational	34	Primary	2021	Treatment- surgery	Other- not specified	N/A
2022	Rubagumya *et al* [101]	Choosing Wisely Africa: insights from the front lines of clinical care	Conquer Cancer; ASCO Foundation	No	No	Retrospective, observational	52	Primary	2021	Research	Other- not specified	N/A
2022	Chasimpha *et al* [88]	Disparities in breast cancer survival between women with and without HIV across sub-Saharan Africa (ABC-DO): a prospective, cohort study	IARC; National Cancer Institute; UK-Commonwealth Scholarships; Susan G Komen	No	No	Retrospective, observational	2,154	Secondary	2014–2017	Epidemiology	Breast	N/A
2022	Mutebi *et al* [10]	Cancer research across Africa: a comparative bibliometric analysis	Wellcome Trust	No	No	Retrospective, observational	23,679	Primary	2009–2020	Research	Other- not specified	N/A
